# The significance of *RB1* in multiple myeloma

**DOI:** 10.3389/fimmu.2024.1415972

**Published:** 2024-11-27

**Authors:** Yiwen Wang, Rui Yang, Rui Liu, Ruoyu Yang, Zujie Lin, Aili He

**Affiliations:** ^1^ Department of Hematology, The Second Affiliated Hospital of Xi’an JiaoTong University, Xi’an, Shaanxi, China; ^2^ Xi’an Key Laboratory of Hematological Diseases, Xi’an, Shaanxi, China

**Keywords:** multiple myeloma, gene mutation, *RB1*, pRB, CDK4/6 inhibitors

## Abstract

The treatment of multiple myeloma (MM) has significantly advanced; however, the underlying genetic mechanisms remain elusive. Clonal events and genetic alterations are recognized as pivotal in the pathogenesis of MM. It is now understood that a multitude of gene mutations, including those affecting RAS, *TP53*, *RB1*, and 1q21 amplification, are prevalent in this disease. The incorporation of several high-risk genetic factors into the Second Revision of the International Staging System (R^2^-ISS) underscores the prognostic significance of genetic aberrations in MM. The retinoblastoma gene (*RB1*), located in 13q14, encodes the retinoblastoma protein (pRB), a tumor suppressor that regulates cell cycle progression. Deletion of *RB1*, which is a frequent event in MM, contributes to tumorigenesis by disrupting cell cycle control. In this respect, *RB1* loss has been implicated in the progression of MM through its influence on interleukin-6 (IL-6) secretion and cell proliferation. This review comprehensively summarizes the role of *RB1* in MM and expounds on the potential of targeting *RB1* as a therapeutic strategy for this malignancy.

## 1 Introduction

Multiple myeloma (MM) is a malignant proliferative neoplasm originating from plasma cells within the bone marrow. Despite significant advancements in therapy that have improved patient outcomes, a comprehensive understanding of the gene mutations and their underlying mechanisms in MM remains crucial for the development of more effective treatments. To date, numerous genes have been implicated in the prognosis of MM, including deletions of chromosomes 13 and 17p (1). Among them, the retinoblastoma susceptibility gene (*RB1*) was the first tumor suppressor gene identified. There is growing consensus suggesting that *RB1* mutations play a critical role in the development of various cancers, including breast and prostate cancer (2, 3). Located on chromosome 13, *RB1* deletions in the 13q14.2 region are frequently associated with tumorigenesis (4). In addition, loss of the retinoblastoma (RB) protein (pRB) has been strongly linked to deletions in the 13q chromosomal region, encompassing *RB1* (5). Notably, *RB1* deletions have been detected in approximately 50% of MM cases, predominantly due to complete monosomy 13 (85%), and are correlated with poorer prognosis (6). Consequently, *RB1* is considered a tumor suppressor gene in MM (7). This review aimed to summarize the function of *RB1* in MM and to explore the potential of targeting *RB1* for MM treatment.

## 2 Roles of cytogenetic abnormalities in the pathogenesis of multiple myeloma

MM is a malignancy derived from plasma cells characterized by the secretion of monoclonal immunoglobulins. Clonal events, which occur in virtually all myeloma cells, including monoclonal gammopathy of undetermined significance (MGUS) and smoldering multiple myeloma (SMM), are pivotal in disease initiation. Acquired genetic alterations such as copy number gains, secondary translocations, and somatic mutations contribute to the progression of myeloma by disrupting cell cycle regulation (8).

Current evidence suggests that abnormal copy numbers can result in the gain or loss of chromosomal regions, contributing to the initiation and progression of malignant processes. Deletions of tumor suppressor genes, including but not limited to *CDKN2C*, *FAF1*, and *FAM46C* [due to del(1p)], *BIRC2* and *NIRC3* [due to del(11q)], *RB1* and *DIS3* [due to del(13q)], and *TP53* [due to del(17p)], can occur through the deletion of the chromosomal arms (q or p). Among these, the *RB1* gene is a critical tumor suppressor with a significant role in the pathogenesis of MM.

Common secondary translocations are simple, reciprocal exchanges that frequently involve breakpoints within or adjacent to the immunoglobulin heavy chain (IgH) switch region. These translocations are believed to arise from aberrant IgH class switch recombination. However, given the infrequent occurrence of IgH class switch recombination in plasma cell tumors and the association of complex *MYC* gene translocations and insertions with advanced disease stages, *MYC* rearrangement is considered a potential mechanism underlying genomic instability. *MYC* rearrangement often positions the *MYC* gene near potent immunoglobulin enhancers, contributing to genomic instability and potentially leading to secondary ectopic insertions (9, 10).

Patients with myeloma frequently harbor somatic mutations within genes of the RAS/MAPK pathway (9). In addition, the pathogenesis of myeloma has been implicated in the dysregulation of the NF-κB and PI3K pathways (11). Moreover, aberrant apoptotic pathways, such as those resulting from the *t*(11;14) translocation leading to BCL2 dependence in malignant plasma cells or MCL1 dependence in other patients, have been proposed to contribute to the development of myeloma (12).

Subsequent acquired genetic events have a significant relationship with the development of MM. Major genetic alterations occur earlier in the disease progression, and the differences in genetic changes between SMM and MM were less pronounced than those between SMM and MGUS. This suggests that additional factors, such as the bone marrow microenvironment, may contribute to disease pathogenesis. MM evolves through a complex interplay between clonal expansion and the bone marrow microenvironment. As the disease progresses, there is an increase of tumor-promoting immune cells and a concomitant loss of anti-tumor immune cells (13).

Currently, the traditional prognostic score for MM is determined using clinical and biochemical parameters, known as the International Staging System (ISS). However, the more recent R^2^-ISS incorporates cytogenetic features, including del(17p) and 1q21 amplification (14, 15). Genomic analysis of patients with MM revealed significant heterogeneity both inter- and intra-patient (16). Moreover, numerous gene mutations have been documented in newly diagnosed patients, with uneven distribution among subclonal units (16). Despite significant advancements in MM treatment, 25% of newly diagnosed patients exhibit a less than 3-year overall survival (17). High-risk cytogenetic abnormalities (HRCAs) warrant specific treatment regimens, such as three or four-drug induction therapy (PI/IMiD/Dex or PI/IMiD/Dex/anti-CD38 antibody), autologous transplantation, and lenalidomide ± PI consolidation/maintenance (18). The increased detection of common gene mutations has improved the clinical risk scores and offered more reliable prognostication (19). These findings underscore the prognostic significance of certain gene mutations and the need for tailored treatment approaches for patients with poor prognoses. While treatment advances have yielded excellent outcomes for some high-risk myeloma patients, a lot of newly diagnosed individuals still experience treatment failure. Therefore, it is imperative to refine the high-risk myeloma definitions, delineate the MM subtypes, and develop more precise, biologically driven treatment strategies. Elucidating the mechanisms underlying the different gene mutations and the pathogenesis of MM is crucial for optimizing patient care.

## 3 Biological characteristics of the *RB1* gene

The *RB1* gene was the first tumor suppressor gene identified, which was originally linked to the development of retinoblastoma (20, 21).

*RB1* is located on chromosome 13q14, spans approximately 180 kb, and comprises 27 exons expressed in most tissues. Studies of *RB1*-related pathways have revealed striking similarities in the DNA sequence characteristics across tumor types with diverse genetic and epigenetic profiles (20). These findings suggest that modulating the *RB1* pathways through targeted interventions based on the biological nature of regulatory *RB1* mutations could offer a promising avenue for precise disease treatment.

**Figure 1:**
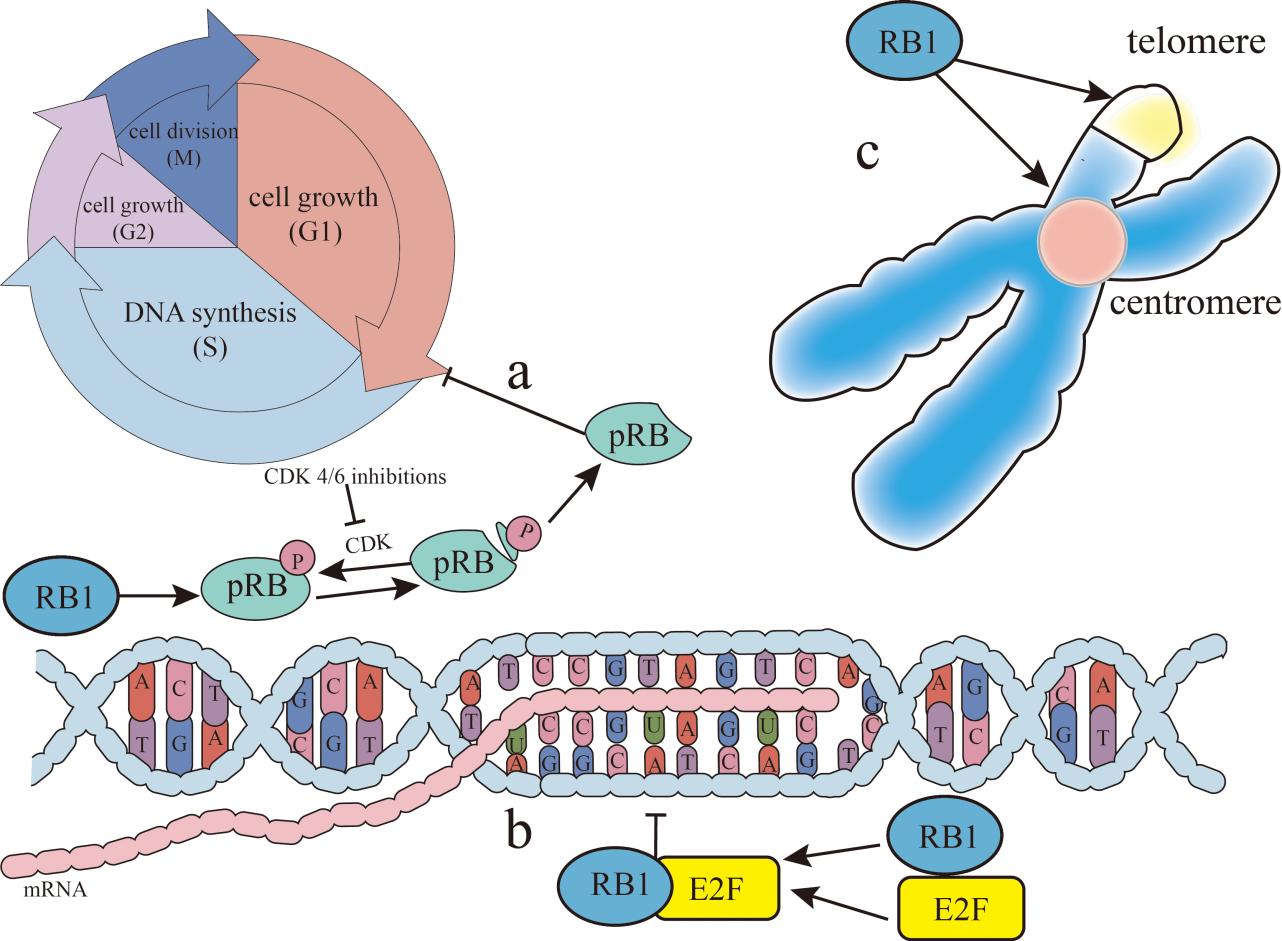
*RB1* (retinoblastoma gene) function. There are three main ways *RB1* can inhibit cancer. **(A)** The retinoblastoma protein (pRB), the gene product of *RB1*, inhibits cancer by dephosphorylation as an active state, blocking the gap 1 (G1)/synthesis (S) phases of the cell cycle. Cyclin-dependent kinase (CDK) can phosphorylate pRB, thereby limiting this process and allowing cancer to progress. CDK4/CDK6 inhibitors can relieve the phosphorylation of pRB, so that pRB can re-inhibit the G1/S phases of the cell cycle to achieve cancer treatment. **(B)** *RB1* inhibits the transcription of the cell cycle genes by binding to early 2 factor (E2F) and regulating the E2F transcription factors. **(C)** *RB1* is associated with the organization and maintenance of heterochromatin, contributing to both stable gene expression for cell differentiation and chromosomal structure at centromeres and telomeres.

*RB1* exerts its tumor suppressor function through three primary mechanisms. Firstly, it regulates early 2 factor (E2F) transcription factors, inhibiting the transcription of cell cycle genes. The pRB–E2F complex further suppresses pluripotent genes, such as *SOX2* in lung cancer (22) and *EZH2* in prostate cancer (23). Secondly, *RB1* contributes to epigenome stability by inhibiting the cell division cycle, allowing for proper epigenome establishment post-replication. Finally, *RB1* is integral to heterochromatin organization and maintenance (24), which are essential for both cell differentiation and chromosomal integrity, including telomere stability. Consequently, *RB1* loss disrupts cell differentiation and compromises chromosome segregation and telomere maintenance (**Figure 1**).

The *RB1* gene encodes pRB, a key regulator of the cell cycle whose function is modulated by phosphorylation. As a multifunctional chromatin-associated protein, pRB negatively regulates cell proliferation by interacting with chromatin regulators to inhibit transcription (25). As a multifunctional chromatin-related protein, pRB can inhibit transcription by interacting with chromatin regulators, stabilizing the complexes that suppress transcription. The hypophosphorylated form of pRB predominantly exists in quiescent cells, while the phosphorylated form appears at the onset of DNA synthesis (26). Dephosphorylation or the inhibition of pRB phosphorylation can trap cells in the gap 1 (G1)/synthesis (S) phases (27), indicating that the hypophosphorylated form is the active state of pRB. During the cell cycle, cyclin-dependent kinase (CDK) phosphorylates pRB, thereby attenuating its activity. The hyperphosphorylation of pRB during the G1/S transition releases its inhibition of E2F, lifting the cell cycle restrictions and initiating processes that can lead to tumorigenesis. In addition, pRB is implicated in chromosome domain organization and gene activation (28). In summary, pRB, the *RB1* gene product, primarily functions as a tumor suppressor through its hypophosphorylated active form, which can arrest the cell cycle. However, mutations in *RB1* or aberrant pRB phosphorylation can disrupt cell cycle control, promoting cancer development or progression. Consequently, the detection of *RB1* and its associated products is critical for prognostic assessment in cancer.

**Figure 2:**
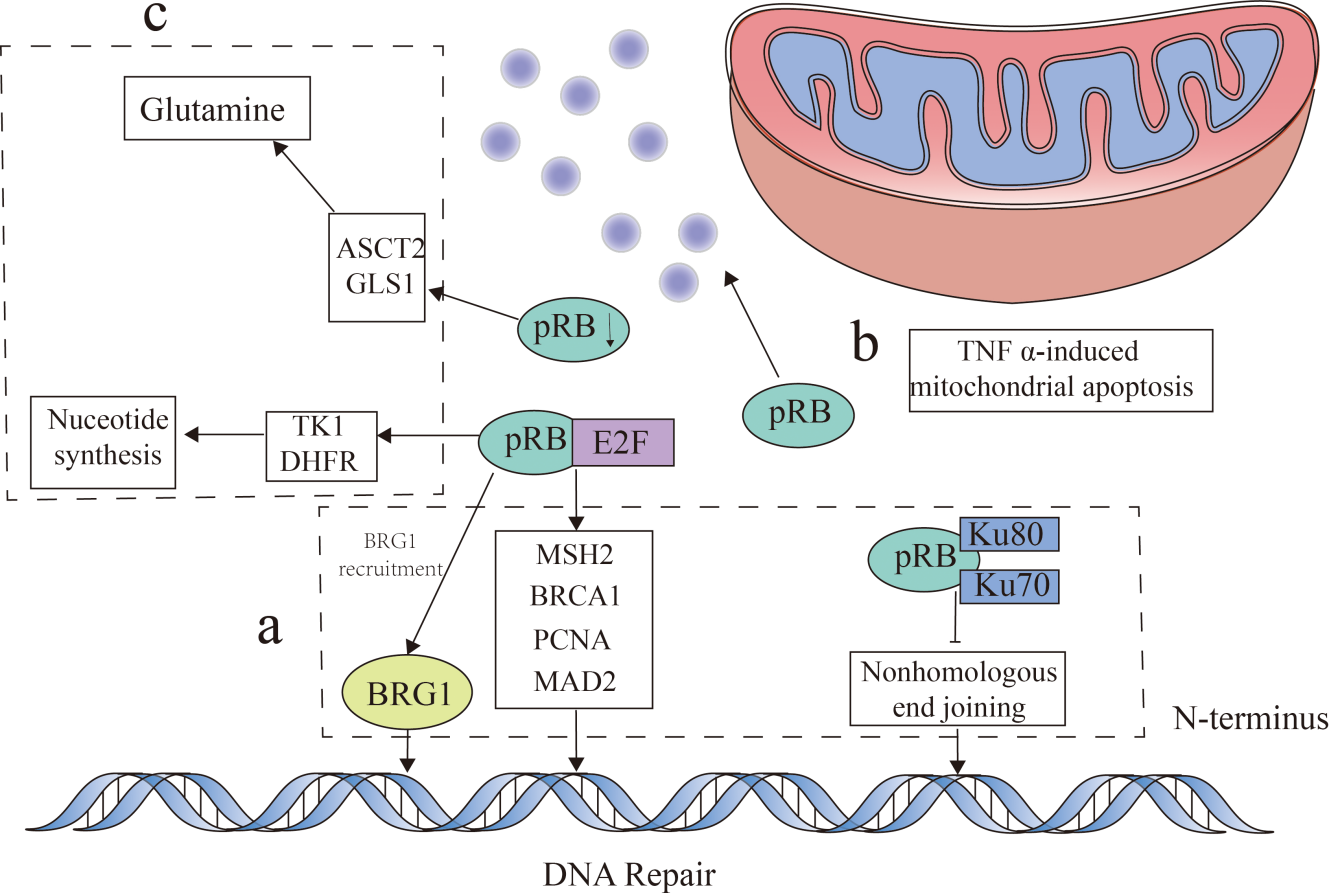
Retinoblastoma protein (pRB) function. pRB exerts its influence through multiple mechanisms. **(A)** This protein regulates DNA damage response by inhibiting early 2 factor (E2F) transcription factors and by modulating the expression of DNA repair factors such as *MSH2*, *BRCA1*, and *PCNA*, as well as facilitating BRG1 recruitment for DNA end repair. In addition, pRB interacts with Ku70 and Ku80 to influence the DNA repair pathways through non-homologous end joining (NHEJ). **(B)** Beyond this, pRB plays a role in the regulation of TNFα-induced mitochondrial apoptosis. **(C)** Moreover, pRB impacts metabolism by inhibiting E2F function, which affects nucleotide biosynthesis through its influence on thymidine kinase (TK1) and dihydrofolate reductase (DHFR). The absence of pRB can lead to the upregulation of the glutamine transporter (ASCT2) and glutaminase (GLS1), consequently affecting glutamine metabolism.

The role of pRB in regulating the cell cycle primarily involves three key aspects (29). Firstly, pRB significantly influences the cell cycle by inhibiting the E2F transcription factors (30). Beyond cell cycle control, pRB also plays a crucial role in DNA damage response by regulating E2F transcription. Loss of pRB leads to E2F activation and the subsequent transcription of target genes, impacting DNA damage by affecting the expression of repair factors such as *MSH2*, *BRCA1*, and *PCNA* (31, 32). In addition, pRB modulates the DNA repair pathways, including non-homologous end joining (NHEJ) and homologous recombination (HR). By interacting with Ku70 and Ku80, pRB facilitates NHEJ-related chromatin modifications (33). In HR, pRB collaborates with E2F to recruit BRG1, a key enzyme in DNA end processing and repair, to double-strand break sites (34). Collectively, these findings highlighted the substantial role of pRB in DNA damage response and repair. Secondly, pRB can induce apoptosis, notably through mitochondrial pathways, by promoting tumor necrosis factor (TNF) activity (35). Furthermore, pRB loss in a mouse bladder cancer model resulted in a decreased p53 expression and the downregulation of apoptotic genes including *BAX*, *BAK*, *BID*, and *APAF1* (36), demonstrating its multifaceted influence on apoptosis. Finally, pRB is essential in metabolic regulation. As mentioned above, pRB inhibits E2F, a key regulator of the cell cycle. Importantly, E2F also participates in numerous metabolic pathways, including nucleotide biosynthesis, glucose oxidation, and mitochondrial function (29). E2F directly promotes nucleotide biosynthesis by upregulating thymidine kinase (TK1) and dihydrofolate reductase (DHFR) (29). In a *Drosophila* model with pRB deficiency, an increased nucleotide metabolism, particularly involving glutamine and glutathione, was observed, suggesting a critical role for pRB in metabolic homeostasis (37). This is further supported by pRB’s upregulation of the glutamine transporter (ASCT2) and glutaminase (GLS1) (38). Moreover, the association of pRB with c-Myc, a transcriptional regulator of metabolic enzymes (39), underscores its significance in metabolic control (**Figure 2**).

During the cell cycle, the CDK-mediated phosphorylation of pRB limits its inhibitory function on E2F, leading to an unchecked cell proliferation. The *RB1* pathway governs the transition from the G1 to the S phase in both mitotic and carcinogenic cell division (30). Stimulated by various factors, including the D-type cyclins, CDK4 and CDK6 become activated and drive this process (27). Their role in facilitating a smooth G1-to-S-phase transition is well established. Moreover, CDK4/CDK6 are pivotal in the uncontrolled cell cycle proliferation characteristic of MM cells (39). These kinases hyperphosphorylate and inactivate pRB, releasing the brakes on cancer cell proliferation and promoting tumorigenesis. The inactivation of *RB1* stimulates downstream gene expression, propelling the cell cycle into the S phase. This process is catalyzed by CDK4 and D1-type cyclins. In contrast, *CDKN2A*, which encodes the p16INK4a protein, acts as a tumor suppressor, opposing CDK4/CDK6 (40). Therapeutic strategies targeting CDK4/CDK6, such as antibodies and inhibitors, offer potential treatment options. By increasing the inhibitory effects of *RB1* on tumor proliferation, CDK4/CDK6 inhibitors have demonstrated efficacy in cancers such as breast cancer (2). Moreover, the inhibition of CDK4/CDK6 has been shown to effectively reduce MM tumor burden (41). Therefore, elucidating the *RB1* pathway and the role of pRB is crucial for understanding the mechanisms of cancer development and for identifying more precise treatment approaches.

## 4 Relationship between *RB1* and MM

Numerous studies have established *RB1* as a frequent cytogenetic abnormality in MM, with deletions affecting approximately 50% of cases and correlating with poor prognosis (6). Karyotype and fluorescence *in situ* hybridization (FISH) analyses of 45 newly diagnosed MM patients in Singapore identified fibroblast growth factor receptor 3 (*FGFR3*) and *RB1* deletions as the most common genetic alterations (42). An investigation on the prognostic factors, including gene variants and copy number variations (CNVs), in newly diagnosed MM patients revealed that loss of 13q is associated with adverse survival outcomes. The prognostic impact was exacerbated when combined with 1q gain, 6p gain, and 13q reduction (43). Furthermore, analysis of patients with MM at different disease stages, from initial diagnosis to end-stage secondary plasmacytic leukemia (sPCL), demonstrated that *RB1* and *ZKSCAN3* mutations emerged exclusively in the final sPCL samples, suggesting a role of *RB1* in both myeloma initiation and extramedullary infiltration (44). In addition, loss of *RB1* has been linked to an increased risk of secondary primary malignancies (SPMs) in patients with MM (7). Chromosome 13 deletions, including microdeletions affecting RB1-critical exons, have been identified in MM cases with *t*(4;14) positivity (7, 45). Moreover, single nucleotide polymorphism (SNP) array and whole-genome sequencing studies have confirmed *RB1* as a recurrent deletion target in MM (44). As a biallelic deletion tumor suppressor gene, the precise role of *RB1* in the pathogenesis of MM and its association with poor prognosis remain to be fully elucidated. A comprehensive understanding of the relationship between *RB1* and MM is essential for accurate prognostication and for the development of effective therapeutic strategies.

### 4.1 RB1 deficiency can increase IL-6 secretion

Interleukin 6 (IL-6) is a cytokine with a pivotal role in inflammation and immunity. It stimulates B-lymphocyte proliferation, differentiation, and antibody production. As early as 1991, studies have explored the potential of blockage of IL-6 signaling for MM treatment (46). While these initial investigations yielded inconclusive results, research into IL-6 antagonists for MM therapy has continued. In myeloma, IL-6-secreting cells can originate from either autocrine or paracrine pathways (47, 48).

IL-6 is a critical factor in myeloma cell proliferation and survival, playing a pivotal role in the pathogenesis of MM. Comparative analysis of IL-6 production between 13q-deficient and non-13q-deficient patients revealed significantly elevated IL-6 levels in the former group. These patients also exhibited resistance to conventional chemotherapy, but sensitivity to thalidomide or bortezomib (49). pRB, the *RB1* gene product, downregulates the IL-6 gene expression, inhibiting MM cell proliferation. *RB1* mutations or pRB alterations lead to IL-6 gene upregulation, contributing to the development and poor prognosis of MM. Numerous IL-6 autocrine and paracrine MM cell lines have been established (50). Studies examining the *RB1* gene transcription and pRB expression in 22 human myeloma cell lines (HMCLs) and in 10 advanced-stage patients did not identify changes in the expression of pRB during the transition from the IL-6 paracrine to autocrine states, suggesting a lack of association between pRB deletion and IL-6 expression in MM. However, the sole HMCL exhibiting an IL-6 autocrine phenotype displayed homozygous *RB1* gene deletion without transcription or expression, implying that the influence of *RB1* on IL-6 secretion and subsequent MM proliferation occurs via an autocrine IL-6 mechanism. In essence, pRB downregulates IL-6 gene expression, and its absence promotes IL-6 autocrine signaling in MM cells, driving malignant proliferation.

Therefore, targeting pRB to suppress IL-6 could potentially represent an effective therapeutic strategy for MM. Currently, tocilizumab, a humanized anti-human interleukin-6 receptor (IL-6R) antibody, is widely used in autoimmune diseases such as rheumatoid arthritis (51). Studies have demonstrated that tocilizumab can bind to COS-7 cells expressing human IL-6R and inhibit the growth of the IL-6-dependent myeloma cell line, KPMM2 (52). These findings suggest that *in vivo* IL-6 reduction may mitigate the progression of MM, although clinical evidence supporting this hypothesis remains limited. In addition, siltuximab, a monoclonal antibody targeting IL-6, was used to treat idiopathic multicentric Castleman disease (iMCD) (53) and cytokine release syndrome (CRS) associated with T-cell redirecting bispecific antibody therapy for relapsed/refractory multiple myeloma (RRMM) (54). While one study indicated a potential delay in high-risk SMM progression with siltuximab (55), overall, research and clinical data on IL-6 reduction as a novel MM treatment strategy are scarce. This paucity of evidence may be attributed to the association between *RB1* deletion and increased IL-6 autocrine signaling in MM. Consequently, simply decreasing the serum IL-6 levels may not be enough to effectively control disease onset and progression. Our findings underscore the potential of targeting *RB1* as a promising therapeutic avenue for MM.

### 4.2 The function of pRB

Retinoblastoma protein, the gene product of *RB1*, is a critical cell cycle regulator. As previously discussed, pRB controls the cell cycle by inhibiting the E2F transcription factors, and its activity is primarily regulated by cyclin-dependent kinases 4 and 6 (CDK4/CDK6). This underscores the pivotal role of pRB as an intermediate within the *RB1* pathway. Immunohistochemical staining and Southern blot analysis of a cohort of MM patients revealed the absence of pRB in 34.7% of advanced cases, suggesting that the inactivation or downregulation of pRB contributes to the progression of MM by disrupting cell cycle control (56). Furthermore, investigations into the cyclin D1/pRb/p16INK4A pathway have linked alterations in cyclin D1 and loss of *RB1* to poor prognosis in MM (57). However, the predominantly non-proliferative nature of myeloma cells presents challenges for studying the expression of pRB using immunostaining methods. This research gap hinders our understanding of the role of pRB in MM and necessitates the development of myeloma cell lines for future investigations. Indeed, pRB is a key player in the progression of MM. Therefore, a comprehensive assessment of not only *RB1* loss but also pRB mutations will provide valuable insights into the pathogenesis of MM.

## 5 Opportunity to use *RB1* targets to treat MM

Current treatments for MM primarily rely on proteasome inhibitors (PIs) and immunomodulatory drugs (IMiDs) (58). However, these therapies are often accompanied by significant side effects, and patient survival remains limited due to the development of drug resistance in many cases (59). Consequently, identifying novel therapeutic approaches for MM represents a critical unmet medical need.

While IL-6 inhibition holds promise as a therapeutic strategy for MM, the efficacy of the current agents targeting IL-6R has been limited in this context. This may be attributed to the observation that the deletion of *RB1* drives tumorigenesis and progression through an IL-6 autocrine loop. Consequently, a mere reduction in the serum IL-6 levels is insufficient for effective MM treatment. Therefore, targeting *RB1* presents a more promising therapeutic avenue.

The *RB1* gene encodes a tumor suppressor protein that inhibits cell proliferation and division. Patients with MM frequently exhibit *RB1* mutations or deletions, leading to tumor cell independence from *RB1* and promoting uncontrolled proliferation and dissemination. Exploiting the mechanism and pathway of *RB1* offers potential therapeutic avenues for MM. During the cell cycle, CDKs hyperphosphorylate pRB, releasing the E2F-mediated cell cycle progression and contributing to tumorigenesis. The *RB1* pathway governs the G1-to-S phase transition in both normal and cancerous cell division (27). D-type cyclins activate CDK4 and CDK6, which subsequently phosphorylate and inactivate the *RB1* tumor suppressor, thereby derepressing cancer cell proliferation. Consequently, inhibiting CDK4/CDK6 can enhance the tumor-suppressive function of *RB1*, as demonstrated in breast cancer treatment (2). CDK4/CDK6 have been implicated in MM cell proliferation, and their inhibition has shown promise in reducing the MM tumor burden (60). However, current clinical trials with approved CDK4/CDK6 inhibitors, such as palbociclib and ribociclib, have shown limited success due to severe hematological toxicities (61–63). Recent studies have identified novel CDK4/CDK6 inhibitors with improved efficacy in inhibiting MM cell proliferation and tumor growth (64). In addition, SET domain bifurcated histone lysine methyltransferase 1 (SETDB1) and the E3 ubiquitin ligase tripartite motif-containing protein 28 (TRIM28) regulate the stability of pRB by inhibiting CDK4/CDK6. TRIM28 promotes the polyubiquitination and degradation of pRB in a phosphorylation-dependent manner, while SETDB1, a chromatin regulator, counteracts the CDK4/CDK6-mediated pRB phosphorylation and subsequent TRIM28-dependent degradation. This suggests a potential therapeutic strategy combining CDK4/CDK6 inhibitors with SETDB1 and TRIM28 modulators to stabilize pRB and suppress tumor growth. Although primarily studied in prostate and breast cancer, this approach warrants investigation in MM (65).

## 6 Conclusion

The deletion of *RB1* is associated not only with the development of MM but also with the occurrence of extramedullary disease. Existing research suggests that the deletion of *RB1* disrupts the downregulation of IL-6 by its gene product, pRB, leading to elevated IL-6 levels and the subsequent malignant proliferation of myeloma cells. However, the predominant IL-6 secretion pathway is currently considered autocrine. Moreover, the deletion of *RB1* contributes to excessive cell proliferation and death. Furthermore, *RB1* deletion likely influences the proliferation of myeloma cells through pRB-mediated cell cycle regulation. While the precise mechanisms underlying the impact of *RB1* deletion on the prognosis and development of MM remain to be fully elucidated, its critical role in this disease is indisputable. A comprehensive understanding of the relationship between *RB1* and MM will facilitate the classification of high-risk MM genetic subtypes and will enable more targeted therapeutic approaches.

## 7 Outlook

Significant advancements in MM treatment have extended overall survival, with some centers reporting a median survival of over 10 years in newly diagnosed patients (66). However, disparities in patient outcomes persist, which are often attributed to the development of drug resistance. Consequently, there is an urgent need for novel therapeutic strategies. To address this unmet clinical need, comprehensive investigations into the relationship between *RB1* loss and MM prognosis are warranted. While extensive research has elucidated the mechanisms underlying the RB family, particularly the role of its gene product, pRB, clinical and mechanistic studies examining the impact of *RB1* on the prognosis of MM remain limited. This gap may be attributed to the complex genetic landscape of MM, which is characterized by multiple gene mutations such as *TP53* loss and 1q amplification. To clarify the association between *RB1* loss and MM prognosis, large-scale studies employing karyotype and FISH analyses are required to identify patient cohorts with *RB1* abnormalities. Subsequently, detailed analyses of these patients can elucidate the prognostic implications of *RB1* loss. Furthermore, functional studies investigating the role of pRB in MM cell lines are essential to understanding the underlying mechanisms. While the importance of CDK4/CDK6 inhibitors in MM has been established, clinical translation remains challenging. Leveraging CDK4/CDK6 inhibitors to further explore the role of *RB1* in MM could provide valuable insights. In addition, considering the potential influence of *RB1* on IL-6 expression and signaling, comprehensive analyses of IL-6 alterations in *RB1*-deficient MM patients are warranted. In conclusion, a more in-depth understanding of the role of *RB1* in the pathogenesis and progression of MM is crucial for the development of targeted therapies. By integrating clinical, genetic, and mechanistic studies, we can advance our knowledge of the impact of *RB1* on the prognosis of MM and identify novel therapeutic opportunities.
